# Effectiveness of emergency surgery for five common acute conditions: an instrumental variable analysis of a national routine database

**DOI:** 10.1111/anae.15730

**Published:** 2022-05-19

**Authors:** A. Hutchings, S. O’Neill, D. Lugo‐Palacios, S. Moler Zapata, R. Silverwood, D. Cromwell, L. Keele, G. Bellingan, S. R. Moonesinghe, N. Smart, R. Hinchliffe, R. Grieve

**Affiliations:** ^1^ Department of Health Services Research and Policy London School of Hygiene and Tropical Medicine London UK; ^2^ Department of Health Services Research and Policy London School of Hygiene and Tropical Medicine London UK; ^3^ Department of Health Services Research and Policy London School of Hygiene and Tropical Medicine London UK; ^4^ University College London UK; ^5^ Department of Health Services Research and Policy London School of Hygiene and Tropical Medicine London UK; ^6^ Clinical Effectiveness Unit Royal College of Surgeons of England London UK; ^7^ University of Pennsylvania Philadelphia PA USA; ^8^ University College London Hospitals NHS Foundation Trust London UK; ^9^ College of Medicine and Health University of Exeter UK; ^10^ Bristol Surgical Trials Centre University of Bristol UK

**Keywords:** abdominal wall hernia, acute appendicitis, diverticular disease, emergency surgery, gallstone disease, intestinal obstruction, surgery

## Abstract

The effectiveness of emergency surgery vs. non‐emergency surgery strategies for emergency admissions with acute appendicitis, gallstone disease, diverticular disease, abdominal wall hernia or intestinal obstruction is unknown. Data on emergency admissions for adult patients from 2010 to 2019 at 175 acute National Health Service hospitals in England were extracted from the Hospital Episode Statistics database. Cohort sizes were: 268,144 (appendicitis); 240,977 (gallstone disease); 138,869 (diverticular disease); 106,432 (hernia); and 133,073 (intestinal obstruction). The primary outcome was number of days alive and out of hospital at 90 days. The effectiveness of emergency surgery vs. non‐emergency surgery strategies was estimated using an instrumental variable design and is reported for the cohort and pre‐specified sub‐groups (age, sex, number of comorbidities and frailty level). Average days alive and out of hospital at 90 days for all five cohorts were similar, with the following mean differences (95%CI) for emergency surgery minus non‐emergency surgery after adjusting for confounding: −0.73 days (−2.10–0.64) for appendicitis; 0.60 (−0.10–1.30) for gallstone disease; −2.66 (−15.7–10.4) for diverticular disease; −0.07 (−2.40–2.25) for hernia; and 3.32 (−3.13–9.76) for intestinal obstruction. For patients with ‘severe frailty’, mean differences (95%CI) in days alive and out of hospital for emergency surgery were lower than for non‐emergency surgery strategies: −21.0 (−27.4 to −14.6) for appendicitis; −5.72 (−11.3 to −0.2) for gallstone disease, −38.9 (−63.3 to −14.6) for diverticular disease; −19.5 (−26.6 to −12.3) for hernia; and − 34.5 (−46.7 to −22.4) for intestinal obstruction. For patients without frailty, the mean differences (95%CI) in days alive and out of hospital were: −0.18 (−1.56–1.20) for appendicitis; 0.93 (0.48–1.39) for gallstone disease; 5.35 (−2.56–13.28) for diverticular disease; 2.26 (0.37–4.15) for hernia; and 18.2 (14.8–22.47) for intestinal obstruction. Emergency surgery and non‐emergency surgery strategies led to similar average days alive and out of hospital at 90 days for five acute conditions. The comparative effectiveness of emergency surgery and non‐emergency surgery strategies for these conditions may be modified by patient factors.

## 1 Introduction

The provision of emergency general surgery raises important challenges to international health care systems and is associated with high morbidity, mortality and resource utilisation [1]. Emergency general surgery provision accounts for approximately 750,000 admissions per year in England alone [2], with surgical procedures representing approximately 10% of the annual National Health Service (NHS) budget [3]. A major concern is that there are wide variations across NHS hospitals in outcomes for patients admitted to emergency general surgery [4]. National professional associations representing surgery [5, 6] and peri‐operative medicine [7] have recognised the importance of improving the efficiency and quality of emergency general surgery provision and have highlighted gaps in the evidence required. National quality improvement programmes such as the National Emergency Laparotomy Audit have helped improve emergency general surgery provision for some common acute conditions [8], focusing on emergency surgery defined as an intervention that is ‘immediate’, ‘urgent’ or ‘expedited’ [9]. However, for some patients, emergency surgery leads to excessive rates of complications and re‐admissions [10].

For acute conditions, such as acute diverticular disease, non‐emergency surgery strategies drawing on multidisciplinary expertise have been developed. These non‐emergency surgery strategies include: medical management; non‐surgical procedures (e.g. radiologically guided drainage of abscess); or surgery deferred to the elective (planned) setting. Over the last 20 years, there has been an increase in the proportion of emergency general surgery admissions for patients over 75 years old, who are more likely to have multiple comorbidities or frailty [11, 12], while emergency surgery rates have declined [12]. Protocols for non‐emergency surgery strategies have been evaluated in randomised controlled trials (RCTs) [13, 14]. For patients with acute appendicitis, one RCT suggested that non‐emergency surgery strategies led to improved outcomes compared with emergency surgery [13], while another reported that outcomes were worse [14]. For patients with acute cholecystitis, RCTs have found that non‐emergency surgery strategies may have unintended consequences with the recurrence of symptoms [15]. However, these RCTs have not included the broad population groups that present as routine emergency general surgery admissions. For example, no RCTs of emergency surgery vs. non‐emergency surgery strategies have been conducted in acute diverticular disease. In observational studies that use administrative datasets, a major concern is that the choice of emergency surgery vs. non‐emergency surgery may be driven by confounders such as disease severity, which may not be measured.

The lack of relevant evidence about the relative effectiveness of emergency surgery vs. non‐emergency surgery strategies for patients with common acute conditions is a major barrier to improving the quality of emergency general surgery services. There is variation in emergency surgery rates across NHS hospitals in England for emergency admissions with common acute conditions [16]. Patients of similar prognosis have emergency surgery in some hospitals and non‐emergency surgery strategies in others. The aim of this study was to assess the relative effectiveness of emergency surgery vs. non‐emergency surgery for five common acute abdominal conditions. The study exploited the natural variation in emergency surgery rates across NHS hospitals in England for these conditions and we have reported relative effectiveness overall and effectiveness according to pre‐specified sub‐groups (age, sex, number of comorbidities and level of frailty).

## 2 Methods

The emergency surgery or not (ESORT) study used Hospital Episodes Statistics data to define patient cohorts admitted as emergencies to 175 NHS acute hospitals for five common acute conditions: appendicitis; symptomatic gall bladder disease; symptomatic diverticular disease; abdominal wall hernia; and intestinal obstruction (small or large bowel) [16]. The patient care database included information on diagnoses, procedures, patients' sociodemographic characteristics and other administrative and organisational information such as dates of admission and discharge [17]. The study protocol and statistical analysis plan were developed following the principles of emulating a target trial and are available on the study website [18, 19].

The research project was approved by the London School of Hygiene and Tropical Medicine ethics committee. The present study involved the secondary analyses of existing pseudo‐anonymised data and did not require UK National Ethics Committee approval. The design and proposed analysis of the ESORT study were informed by a patient and public advisory group during two online workshops held in July 2020 [20]. Workshop participants were asked to consider outcome measures for patients following emergency admission to the hospital for acute conditions, and the group agreed that an appropriate measure would capture both mortality and the number of days in hospital. This group will coproduce a lay summary based on the study findings that will be made available on the ESORT study website (www.lshtm.ac.uk/research/centres‐projects‐groups/esort). Full details of the original study design are available from a previous publication [16] and from the study website [21].

For patients aged 18 years old or older, hospital admissions were eligible if a finished consultant episode within the admission met all the following criteria: occurred between April 2010 and December 2019; included a main diagnosis with an International Classification of Diseases, tenth revision (ICD‐10) diagnosis code judged relevant according to the consensus of a clinical panel (online Supporting Information Appendix S1, Section S1) [21]; was within an emergency admission through the emergency department or from a primary care referral; was under a consultant general surgeon, subspecialty general surgeon or any other surgeon working in the general surgery specialty; and was the first or second episode within the admission. For the intestinal obstruction cohort, a relevant diagnosis could appear in the second diagnosis field if the main diagnosis was colorectal cancer. Admissions for which there was a prior emergency admission with a relevant diagnosis in the previous 12 months were excluded. Online Supporting Information Tables S1 and S2 report the numbers of patients excluded and the final list of diagnostic categories included, respectively.

Emergency surgery was defined from a list of Office of Population Censuses and Surveys codes with a maximum time window, according to the consensus of a clinical panel (online Supporting Information Appendix S1, Section S2) [21]. The qualifying emergency procedure had to be within 3 days (hernia), 7 days (appendicitis, gallstone disease and intestinal obstruction) or any time within the emergency admission (diverticular disease). The non‐emergency surgery strategy was defined as clinical management that did not meet the criteria for emergency surgery. The non‐emergency surgery strategy could include drugs, such as antibiotic therapy, and non‐operative procedures including interventional radiology. Non‐emergency could also include operative procedures that did not meet the criteria for emergency surgery, either because they were not considered a qualifying emergency surgery procedure, were undertaken after the maximum time window for emergency surgery or both. The emergency surgery group could have similar aspects of clinical management to the non‐emergency surgery group, in addition to the emergency surgery strategies. For patients with acute diverticular disease, an example of an emergency surgery strategy was colectomy, while an example of a non‐emergency surgery strategy was percutaneous drainage.

Information was extracted from the database on each patient's clinical management, according to codes for operative and non‐operative procedures within the initial emergency admission and subsequent re‐admissions. For some non‐emergency surgery strategies, for example antibiotic management with no operative procedure, information was not available to define the strategy from the database.

The following baseline characteristics of patients were extracted from the database at admission and were considered to potentially influence the decision as to whether the patient had emergency surgery or a non‐emergency strategy: age (y); sex, ethnicity; index of multiple deprivation (a socio‐economic measure); diagnostic subcategories; number of comorbidities; and frailty. The Charlson comorbidity index [22] and secondary care administrative records frailty index [23] were derived for all patients. This frailty index is based on the accumulation of deficits across a number of domains. It uses ICD‐10 codes to define 32 deficits that cover functional impairment, geriatric syndromes, problems with nutrition, cognition and mood and medical comorbidities. It uses four categories (‘fit’ or mild, moderate or severe frailty) with severe frailty defined as the presence of six or more deficits [23].

The primary outcome measure was the number of days alive and out of hospital (DAOH) at 90 days. Days alive and out of hospital is a composite measure, which encompasses mortality and total length of hospital stay (LOS) including re‐admissions for re‐interventions. It has been recommended as a standardised patient‐centred outcome measure and a core outcome measure for clinical effectiveness trials in peri‐operative medicine [24]. This outcome measure has been formally validated in multiple studies following emergency surgery [25] and was supported by a panel of ex‐patients and public contributors [20]. Days alive and out of hospital was measured from the date the index episode started for up to 90 days, using data on the total duration of hospitalisation over the 90‐day period and the date of death from linkage to the Office for National Statistics death record. For patients who were alive at 90 days, DAOH was calculated by subtracting their total LOS across all admissions across the 90‐day follow‐up period. Those patients who died within the 90‐day period were assigned zero DAOH [24]. The study's sample size for each condition was projected to be sufficient to assess overall differences between the comparison groups in the mean DAOH of at least 3 days, with 80% power, and 95% levels of statistical significance [19]. A difference in mean DAOH of this magnitude has been judged to be of clinical significance [26]. The secondary outcomes were: 90‐day mortality; total LOS before 90 days (these first two outcomes made up the DAOH composite measure); and any emergency re‐admission within 30 days.

An instrumental variable analysis (online Supporting Information Appendix S1, Section 3) aims to approximate the random assignment of treatment in a RCT, by using an instrument to balance observed and unobserved baseline prognostic measures between the comparison groups [27]. We adopted an instrumental variable previously used to evaluate emergency surgery from USA claims data [28], following pharmaco‐epidemiological research in using clinician preference as an instrument for treatment receipt [29]. In the ESORT study, the instrumental variable was the hospital's tendency to operate (TTO), which reflects practice variation across hospitals in emergency surgery rates for these five conditions (online Supporting Information Figure S1). Unlike the precedent study in the USA, which defined TTO at the surgeon level [28], the ESORT study defined TTO at the hospital level. The hospital‐level TTO was judged to appropriately reflect decision‐making within the NHS, as the decision of emergency surgery vs. non‐emergency surgery strategy reflects multidisciplinary assessment and is more likely to differ across rather than within hospitals. There was also a greater quality assurance about the requisite data at the level of the hospital than at the level of the individual consultant surgeon. For each qualifying emergency admission, the TTO was defined as the proportion of eligible emergency admissions in that specific hospital that received emergency surgery in the previous 12 months, thus suggesting that the hospital's past preference for emergency surgery would strongly predict treatment choice for the current patient.

The rationale for the instrumental variable design was that, after adjustment for observed characteristics, patients' baseline prognoses were similar across hospitals with different levels of TTO. Hence, the patients could be ‘randomised’ between the emergency surgery and non‐emergency surgery strategies according to the hospital's TTO. A valid instrument must meet two conditions [27]. First, the instrument must be associated with the treatment received, with methods guidance requiring that the accompanying F statistic exceeds 10 [30]. Second, the instrument should have no relation to the outcome, except through the treatment. There was no empirical approach to assess whether an instrument was directly associated with an outcome, but we examined the extent to which observed characteristics were balanced across different levels of the instrument as this increased confidence that unobserved confounders were also balanced [31].

The study reported absolute risk differences (binary measures) and difference in means (continuous measures) between the comparison groups. The instrumental variable analysis estimated the relative effectiveness of emergency surgery vs. non‐emergency surgery for each individual and fully accounted for heterogeneity of effects as well as confounding (online Supporting Information Appendix S1, Section 4) [32, 33, 34]. These person‐level treatment effects were aggregated to report the effects of emergency surgery overall and for each pre‐specified sub‐group of interest: age (y); sex; ethnicity; index of multiple deprivation; diagnostic subcategories; Charlson comorbidity index; frailty index; and year of admission. The choice of models for this instrumental variable approach followed methodological guidance [32, 33] using probit regression models to estimate the initial (binary) propensity score (first stage) and generalised linear models to accommodate whether each endpoint was continuous or binary (second stage). The second stage (outcome) models also included adjustment for proxies for the quality of acute hospital care, which were defined as the rates of emergency admission and mortality in each acute hospital from 2009 to 2010 (time constant) and for the year before the admission concerned (time‐varying). Each of these measures of the quality of care was defined for emergency admissions for each acute hospital that met the above eligibility criteria, and these were defined as the raw (unadjusted) variables. Models at both stages also adjusted for the case‐mix measures described above and time period (online Supporting Information Appendix S1, Section 5). The estimates were reported with bootstrapped confidence intervals (300 replications) that allowed for the clustering of individuals within hospitals.

Sensitivity analyses (online Supporting Information Appendix S1, Section 6) were undertaken to assess whether the results were robust to alternative definitions and assumptions. First, stricter criteria for emergency surgery were applied by increasing the required level of clinical panel support, from at least 50% to 75%, for defining a procedure as emergency surgery and for stipulating the maximum time window for emergency surgery. Second, the emergency surgery time windows were reduced by taking the threshold as the upper quartile value from the distribution of the ‘time to emergency surgery’ used in the main analysis. Third, the study adjusted for ‘quality of care’ using external hospital performance measures from the National Emergency Laparotomy Audit [8]. Fourth, the study excluded observations from hospitals with low volumes of emergency surgery procedures (at least one interquartile range below the median). Fifth, the DAOH measure was changed to ‘count’ days out of hospital for patients who died before 90 days [35]. Sixth, the case‐mix adjustment used logistic regression (binary outcomes) and ordinary least‐squares regression (continuous outcomes), which assumed no unobserved confounding after adjusting for the above baseline characteristics.

## 3 Results

Baseline characteristics of patients are shown in Table 1. The number of emergency admissions included for each category was: 268,144 (appendicitis); 240,977 (gallstone disease); 138,869 (diverticular disease); 106,432 (hernia); and 133,073 (intestinal obstruction). The proportions of patients who had emergency surgery were: 92.3% (acute appendicitis); 21.6% (gallstone disease); 11.4% (diverticular disease); 58.8% (abdominal wall hernia); and 30.5% (intestinal obstruction). Among patients with acute appendicitis or gallstone disease, those in the emergency surgery group were on average younger and less likely to be frail than those in the non‐emergency surgery group. Patients with acute diverticular disease or intestinal obstruction were of similar average age across the groups, with the emergency surgery group more likely to be frail.

**Table 1:** Baseline characteristics of patients of the five cohorts by emergency surgery and non‐emergency surgery groups. Values are number (proportion) or mean (SD).

	Appendicitis n = 268,144		Gallstone disease n = 240,977		Diverticular disease n = 138,869		Hernia n = 106,432		Intestinal obstruction n = 133,073	
	Emergency surgery n = 247,506 (92.3%)	Non‐emergency surgery n = 20,638 (7.7%)	Emergency surgery n = 52,004 (21.6%)	Non‐emergency surgery n = 188,973 (78.4%)	Emergency surgery n = 15,772 (11.4%)	Non‐emergency surgery n = 123,097 (88.6%)	Emergency surgery n = 62,559 (58.8%)	Non‐emergency surgery n = 43,873 (41.2%)	Emergency surgery n = 40,550 (30.5%)	Non‐emergency surgery n = 92,523 (69.5%)
Sex; female	113,224 (45.8%)	10,228 (49.6%)	36,864 (70.9%)	125,927 (66.6%)	8698 (55.2%)	73,172 (59.4%)	25,035 (40.0%)	12,530 (28.6%)	23,269 (57.4%)	46,708 (50.5%)
Age; y	38.3 (16.3)	47.3 (20.2)	50.7 (17.7)	56.1 (19.2)	63.9 (14.8)	64.0 (15.7)	63.1 (18.2)	62.2 (19.3)	66.6 (16.5)	67.8 (17.2)
Charlson comorbidity index										
0	207,525 (83.9%)	15,321 (74.2%)	36,737 (70.6%)	120,748 (63.9%)	9789 (62.1%)	73,457 (59.7%)	39,216 (62.7%)	26,297 (59.9%)	22,487 (55.5%)	47,161 (51.0%)
1	35,721 (14.4%)	3989 (19.3%)	12,287 (23.6%)	49,863 (26.4%)	4482 (28.4%)	35,106 (28.5%)	17,494 (28.0%)	12,163 (27.7%)	12,849 (31.7%)	29,296 (31.7%)
2	3715 (1.5%)	1035 (5.0%)	2544 (4.9%)	14,503 (7.7%)	1222 (7.8%)	11,454 (9.3%)	4792 (7.7%)	4169 (9.5%)	4221 (10.4%)	12,425 (13.4%)
3+	545 (0.2%)	293 (1.4%)	436 (0.8%)	3859 (2.0%)	279 (1.8%)	3080 (2.5%)	1057 (1.7%)	1244 (2.8%)	993 (2.5%)	3641 (3.9%)
SCARF frailty index										
Fit	206,796 (83.6%)	15,015 (72.8%)	34,056 (65.5%)	114,973 (60.8%)	6197 (39.3%)	65,911 (53.5%)	33,014 (52.8%)	23,871 (54.4%)	17,473 (43.1%)	42,879 (46.3%)
Mild frailty	34,544 (14.0%)	4052 (19.6%)	13,608 (26.2%)	52,629 (27.9%)	5631 (35.7%)	38,851 (31.6%)	19,608 (31.3%)	13,104 (29.9%)	13,722 (33.8%)	30,286 (32.7%)
Moderate frailty	5041 (2.0%)	1155 (5.6%)	3385 (6.5%)	16,175 (8.6%)	2706 (17.2%)	13,433 (10.9%)	7360 (11.8%)	4987 (11.4%)	6511 (16.1%)	13,493 (14.6%)
Severe frailty	1125 (0.5%)	416 (2.0%)	955 (1.8%)	5196 (2.8%)	1238 (7.9%)	4902 (4.0%)	2577 (4.1%)	1911 (4.4%)	2844 (7.0%)	5865 (6.3%)

SCARF, secondary care administrative records frailty.

The TTO instrumental variable was judged to meet each of the requisite criteria for validity. First, the hospital's TTO was strongly correlated with emergency surgery receipt, with F‐statistics that ranged from 141 (appendicitis) to 9053 (gallstone disease), vs. the requirement that they exceed 10 (online Supporting Information Table S3). Second, the hospital's TTO balanced baseline covariates (online Supporting Information Figure S2).

Table 2 reports the most common surgical procedures in the emergency surgery group. For patients with acute appendicitis or gallstone disease, the majority of patients had emergency excision of the appendix (93.5%) or total cholecystectomy (85.3%), respectively. For patients with diverticular disease, the most common form of emergency surgery was end colostomy (Hartmann's procedure) (44.1%). For patients with abdominal wall hernia, the proportions having repairs of inguinal (40.3%), umbilical (36.3%) and femoral (19.5%) hernias reflected the respective diagnostic subcategories (online Supporting Information Table S2). The proportions of patients in the non‐emergency surgery strategy groups who had operative procedures after the time window but within 30 days were < 5% for all conditions (online Supporting Information Table S4). The proportion of patients who had abdominal interventional radiology procedures was low and similar across emergency surgery and non‐emergency surgery groups. For patients with acute appendicitis, the proportion of patients who had imaging or diagnostic procedures was higher for the non‐emergency surgery vs. emergency surgery strategy group.

**Table 2:** Most common surgical procedures in the emergency surgery group. Values are median (IQR [range]) or number (proportion).

	Appendicitis (n = 247,506)	Gallstone disease (n = 52,004)	Diverticular disease (n = 15,772)	Hernia (n = 62,559)	Intestinal obstruction (n = 40,550)
Days to surgery	1 (0–1 [0–7])	2 (1–4 [0–7])	1 (0–2 [0–68])	0 (0–1 [0–3])	1 (0–3 [0–7])
Common main procedures	Emergency excision of the appendix 231,418 (93.5%) Appendicectomy and endoscopic resection of lesion of peritoneum 7425 (3.0%) Emergency excision of the appendix and drainage 4702 (1.9%) Other 3961 (1.6%)	Total cholecystectomy 44,578 (85.7%) Drainage of gall bladder 3640 (7.0%) Partial cholecystectomy 1753 (3.4%) Total cholecystectomy exploration bile duct 1285 (2.5%) Other 748 (1.4%)	Resection with end colostomy ‐ Hartmann's 6955 (44.1%) Resection with other colostomy (e.g. loop colostomy) 4053 (25.7%) Irrigation/drainage (colon, abdominal or pelvic area) 2003 (12.7%) Resection and anastomosis 1088 (6.9%) Colostomy with no resection on the same date 451 (2.9%) Other 1222 (7.7%)	Repair of inguinal hernia 24,955 (39.9%) Repair of umbilical hernia 23,440 (37.5%) Repair of femoral hernia 12,066 (19.3%) Repair of ventral hernia 1642 (2.6%) Other 456 (0.7%)	Freeing of adhesions of peritoneum and related procedures 20,825 (51.4%) Hemicolectomy 5052 (12.5%) Colostomy or Ileostomy 2788 (6.9%) Ileectomy 4129 (10.2%) Rectosigmoidectomy and related procedures 2028 (5.0%) Hernia repair 487 (1.2%) Other 5241 (12.9%)

Table 3 presents absolute mean differences between the emergency surgery and non‐emergency surgery strategy groups, before adjusting for case‐mix differences. For patients with diverticular disease, the mean DAOH at 90 days was 19 days lower in the emergency surgery vs. non‐emergency surgery strategy groups, and a higher proportion of patients in the emergency surgery group died before 90 days (online Supporting Information Figure S3). For the other four conditions, the unadjusted differences in the mean DAOH were relatively small.

**Table 3:** Unadjusted outcomes following emergency surgery and non‐emergency surgery strategies. Values are mean (SD) or number (proportion) with absolute mean differences (95%CI) for emergency surgery vs. non‐emergency surgery.

	Emergency surgery	Non‐emergency surgery	Mean difference (95%CI)
Appendicitis			
DAOH; days within 90 days (mean)	84.78 (6.07)	82.50 (11.41)	2.28 (2.01–2.54)
Mortality within 90 days	449 (0.19%)	219 (1.09%)	−0.90 (−1.07 to −0.73)
LOS; days within 90 days (mean)	5.09 (4.92)	6.68 (7.59)	−1.60 (−1.77 to −1.42)
Emergency re‐admissions within 30 days	21,941 (9.06%)	2237 (11.09%)	−2.03 (−2.55 to −1.51)
Gallstone disease			
DAOH; days within 90 days (mean)	81.28 (10.45)	80.74 (13.14)	0.54 (0.22–0.87)
Mortality within 90 days	372 (0.73%)	2781 (1.50%)	−0.77 (−0.9 to −0.64)
LOS; days within 90 days (mean)	8.23 (8.04)	8.22 (8.89)	0.01 (−0.26–0.28)
Emergency re‐admissions 30 days	5440 (10.63%)	26,807 (14.43%)	−3.80 (−4.23 to −3.37)
Diverticular disease			
DAOH; days within 90 days (mean)	60.92 (26.23)	79.94 (16.71)	−19.0 (−19.6 to −18.4)
Mortality within 90 days	1396 (9.30%)	3690 (3.04%)	6.27 (5.74–6.79)
LOS; days within 90 days (mean)	22.38 (18.37)	7.84 (9.42)	14.60 (14.2–14.9)
Emergency re‐admissions within 30 days	1326 (8.84%)	11,810 (9.72%)	−0.88 (−1.42–0.35)
Hernia			
DAOH; days within 90 days (mean)	80.98 (16.57)	81.63 (18.29)	−0.65 (−0.95 to −0.35)
Mortality within 90 days	1629 (2.68%)	1596 (3.69%)	−1.01 (−1.25 to −0.78)
LOS; days within 90 days (mean)	7.08 (10.19)	5.49 (9.32)	1.59 (1.42–1.76)
Emergency re‐admissions within 30 days	5735 (9.43%)	5360 (12.40%)	−2.97 (−3.38 to −2.56)
Intestinal obstruction			
DAOH; days within 90 days (mean)	66.56 (24.25)	68.01 (29.74)	−1.46 (−2.00 to −0.91)
Mortality within 90 days	3011 (7.59%)	12,155 (13.37%)	−5.78 (−6.33 to −5.22)
LOS; days within 90 days (mean)	18.13 (15.68)	11.80 (13.97)	6.33 (6.07–6.58)
Emergency re‐admissions within 30 days	3770 (9.50%)	13,388 (14.72%)	−5.22 (−5.61 to −4.82)

DAOH, days alive and out of hospital; LOS, length of stay.

Table 4 presents the results from the instrumental variable analysis. There were small overall mean differences (95%CI) in DAOH at 90 days between the emergency surgery vs. non‐emergency surgery strategy groups (emergency surgery minus non‐emergency surgery): −0.73 days (−2.10–0.64) for appendicitis; 0.60 (−0.10–1.30) for gallstone disease; −2.66 (−15.7–10.4) for diverticular disease; −0.07 (−2.40–2.25) for hernia; and 3.32 (−3.13–9.76) for intestinal obstruction. The instrumental variable analysis also reported that, for four of the conditions, the effect of emergency surgery vs. non‐emergency surgery strategies on 90‐day mortality was small and not statistically significant. For patients with abdominal wall hernia, absolute risk of death favoured emergency surgery strategies with a mean difference (95%CI) of −4.99 (−9.92 to −0.07) percentage points compared with non‐emergency surgery strategies. Hospital LOS was longer following emergency surgery vs. non‐emergency surgery strategies for abdominal wall hernia (mean difference 2.35 days) and was shorter for intestinal obstruction (mean difference 4.25 days). The emergency surgery strategies led to reductions in the proportion of emergency re‐admissions before 30 days, compared with the non‐emergency surgery strategies for all the conditions apart from acute appendicitis.

**Table 4:** Effects of emergency surgery vs. non‐emergency surgery strategies on days alive and out of hospital, all‐cause mortality, length of stay and emergency re‐admissions from instrumental variable analysis.

	Mean difference	95%CI	p‐value
Appendicitis			
DAOH; days within 90 days (mean)	−0.73	(−2.10–0.64)	0.30
Mortality within 90 days (%)	0.24	(−0.04–0.51)	0.09
LOS; days within 90 days (mean)	0.03	(−1.04–1.10)	0.96
Emergency re‐admissions within 30 days (%)	−2.50	(−10.3–5.26)	0.53
Gallstone disease			
DAOH; days within 90 days (mean)	0.60	(−0.10–1.30)	0.09
Mortality within 90 days (%)	0.18	(−0.97–1.32)	0.76
LOS; days within 90 days (mean)	−0.43	(−0.88–0.03)	0.07
Emergency re‐admissions within 30 days (%)	−3.85	(−5.54 to −2.16)	< 0.01
Diverticular disease			
DAOH; days within 90 days (mean)	−2.66	(−15.7–10.4)	0.69
Mortality within 90 days (%)	3.34	(−5.22–11.9)	0.45
LOS; days within 90 days (mean)	2.28	(−5.23–9.80)	0.55
Emergency re‐admissions within 30 days (%)	−12.6	(−21.4 to −3.77)	< 0.01
Hernia			
DAOH; days within 90 days (mean)	−0.07	(−2.40–2.25)	0.95
Mortality within 90 days (%)	−4.99	(−9.92 to −0.07)	0.05
LOS; days within 90 days (mean)	2.35	(1.54–3.15)	< 0.01
Emergency re‐admissions within 30 days (%)	−4.05	(−7.77 to −0.33)	0.03
Intestinal obstruction			
DAOH; days within 90 days (mean)	3.32	(−3.13–9.76)	0.31
Mortality within 90 days (%)	1.73	(−4.93–8.38)	0.61
LOS; days within 90 days (mean)	−4.25	(−7.50 to −1.00)	0.01
Emergency re‐admissions within 30 days (%)	−8.62	(−18.1–0.88)	0.08

DAOH, days alive and out of hospital; LOS, length of stay.

**Figure 1:**
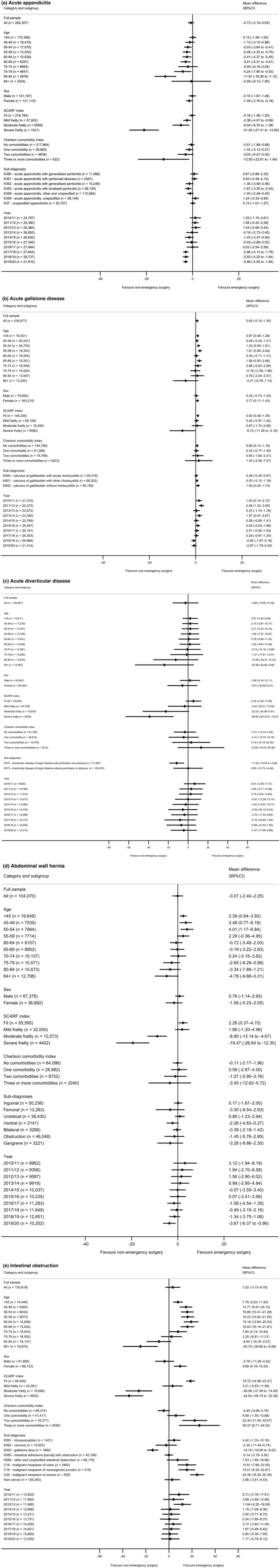
Effectiveness of emergency surgery vs. non‐emergency surgery strategies on DAOH up to 90 days by sub‐group. a, acute appendicitis; b, acute gallstone disease; c, acute diverticular disease; d, abdominal wall hernia; e, intestinal obstruction. SCARF index, secondary care administrative records frailty index.

The relative effectiveness of emergency surgery vs. non‐emergency surgery strategies was modified by age group, with emergency surgery less effective for some sub‐groups of older patients (Fig. 1a‐e). For patients aged 80–84 y and 85 y or older, the respective differences in the mean DAOH following emergency surgery vs. non‐emergency surgery were: appendicitis −11.81 and −0.58; gallstone disease 0.76 and −4.31; diverticular disease −12.49 and −23.98; hernia −3.34 and −4.78; and intestinal obstruction −24.7 and −8.64. Conversely, emergency surgery was more effective than non‐emergency surgery in some younger age groups for gallstone disease, hernia and intestinal obstruction.

For patients with three or more comorbidities, mean difference (95%CI) in DAOH at 90 days associated with emergency surgery strategies was lower for patients with acute appendicitis (−12.55 (−23.61 to −1.49)); higher for patients with intestinal obstruction (26.37 (8.71–44.02)); and similar between the comparison groups for the other three conditions.

For patients with severe frailty, the mean DAOH at 90 days following emergency surgery was lower than after non‐emergency surgery strategies, with mean differences (95%CI) for appendicitis of −21.0 (−27.4 to −14.6); gallstone disease −5.72 (−11.3 to −0.2); diverticular disease −38.9 (−63.3 to −14.6); hernia −19.5 (−26.6 to −12.3); and intestinal obstruction −34.5 (−46.7 to −22.4). For patients without frailty, the mean difference (95%CI) in DAOH favoured emergency surgery for hernia (2.26 (0.37–4.15)) and intestinal obstruction (18.2 (14.8–22.47)).

For patients with severe frailty, there was an increase in 90‐day mortality following emergency surgery vs. non‐emergency surgery strategies for all conditions apart from hernia (online Supporting Information Figure S4), and there was an increase in mean LOS for emergency surgery vs. non‐emergency surgery strategies for all conditions (online Supporting Information Figure S5). For each condition, the estimates of the effectiveness of emergency surgery vs. non‐emergency surgery for each year of admission were consistent with the overall estimates.

The overall effects of the emergency surgery vs. non‐emergency surgery strategies were similar in sensitivity analyses when alternative standpoints were considered (online Supporting Information Figure S6). Specifically, when alternative, more stringent criteria for the definitions of emergency surgery were used, the mean differences in DAOH remained below 4 days for each of the conditions. The overall results were also robust to the use of alternative definitions of DAOH and the ‘quality’ of acute care and to the exclusion of observations from hospitals with low volumes of emergency surgery. The regression analysis found that emergency surgery was associated with an average reduction in DAOH for patients with diverticular disease but did not take into account unobserved confounding.

## 4 Discussion

For emergency general surgery admissions with acute appendicitis, gallstone disease, diverticular disease, abdominal wall hernia or intestinal obstruction, this study found that the average number of DAOH at 90 days was similar following emergency surgery and non‐emergency surgery strategies. There were differences in the relative effectiveness of emergency surgery vs. non‐emergency surgery strategies, according to patients' levels of frailty, age and number of comorbidities. For patients with severe frailty, average DAOH was lower, and mean total LOS was higher, following emergency surgery vs. non‐emergency surgery strategies for all five conditions. For patients with severe frailty, all‐cause mortality was higher at 90 days following the emergency surgery vs. non‐emergency surgery strategies for patients with acute appendicitis, diverticular disease and intestinal obstruction. For patients without frailty, emergency surgery strategies were more effective for patients with hernia and intestinal obstruction. These findings have implications for emergency general surgery provision and emphasise the importance of providing evidence‐based care tailored to patient factors such as frailty, number of comorbidities and age.

This study adds to our understanding of the effectiveness of emergency surgery vs. non‐emergency surgery strategies such as delayed surgery, interventional radiology or medical management for common acute abdominal conditions. Randomised controlled trials have reported equivocal results for selected patients with uncomplicated acute appendicitis [13, 14]. The study by Javanmard‐Emamghissi et al. exploited the increased rates of non‐operative strategies following the first wave of COVID‐19 infections and reported that non‐emergency surgery strategies led to short‐term cost savings [36]. For patients presenting with acute cholecystitis, published guidelines recommend laparoscopic cholecystectomy within 7 days of diagnosis [37], but emergency surgery rates vary across NHS hospitals [16]. For patients with acute diverticular disease, published RCTs have been underpowered to truly evaluate emergency surgery vs. non‐emergency surgery strategies [38, 39]. For patients with abdominal wall hernia or intestinal obstruction presenting as emergency admissions, there are no published RCTs comparing emergency surgery and non‐emergency surgery strategies. Hence, the findings from the present study (that emergency surgery and non‐emergency surgery strategies led to similar average DAOH at 90 days across unselected patients routinely presenting as emergency admissions) contribute new knowledge to inform emergency general surgery provision.

The ESORT study findings support guidance from national associations representing peri‐operative medicine and surgery [6, 7], that encourage consideration of factors beyond biological age such as frailty and patient comorbidities when deciding on the timing of operative procedures. This guidance emphasises the importance of careful peri‐operative assessment by a multidisciplinary team before deciding on emergency or non‐emergency surgery strategies, to help recognise the potential advantages of delayed surgery in reducing risks of medical complications for patients with severe frailty or multiple comorbidities [11, 40, 41]. We found that non‐emergency surgery strategies were more effective for patients with severe frailty (up to 8% of each cohort). The higher mean DAOH following non‐emergency surgery were partly driven by reduced all‐cause mortality at 90 days (except for hernia) and reduced LOS. Randomised controlled trials of emergency surgery strategies have either excluded frail patients or not considered whether frailty modifies the relative effectiveness of emergency surgery vs. non‐emergency surgery [13, 14, 15]. This study found that, for sub‐groups including patients with hernia and intestinal obstruction who were classified as ‘fit’ (39–84% of the cohorts), emergency surgery strategies increased the average DAOH. The current study therefore supports guidelines that emphasise the importance of peri‐operative frailty assessment even in the emergency setting [7].

This study has several strengths and limitations. It included a sufficient number and range of patients with these common acute conditions to provide generalisable and precise estimates of the comparative effectiveness of emergency surgery vs. non‐emergency surgery strategies as provided in routine practice. The study used a previously developed instrumental variable method to address confounding and provide comparative effectiveness estimates that apply to the overall populations and subpopulations of interest. The limitations of this paper are that detailed information from imaging or diagnostic procedures was unavailable; therefore, the definitions of sub‐groups were broad. The categorisation of emergency surgery vs. non‐emergency surgery strategies assumes accurate coding of procedures and episode dates. While it is conceivable that there were coding differences across NHS hospitals or over time, it is unlikely that this led to substantive differences in the estimates of relative effectiveness. The primary outcome measure, DAOH, did not consider dimensions of health, such as health‐related quality of life or complication rates, which were not available within the database. The definition of non‐emergency surgery strategies was limited to procedure codes and could not capture other aspects of clinical management (e.g. type and duration of antibiotic therapy). The ability of this study to draw causal inference from a retrospective cohort study rests on the instrumental variable approach to deal with unmeasured confounding and may be undermined if the requisite assumptions do not hold. However, the finding that the instrumental variable balanced important case‐mix measures, such as frailty and comorbidity, provides assurance that it would also ensure that indicators of disease severity that are not available in the database were also similar between the emergency surgery and non‐emergency surgery groups.

This study highlights various areas for further research. For patients with abdominal wall hernia, the ESORT study found similar rates of emergency vs. non‐emergency surgery strategies, and outcomes for most sub‐groups were similar for patients who had emergency surgery vs. those who did not. Hence, the ESORT study findings suggest that a RCT contrasting emergency with non‐emergency surgery strategies could be warranted for patients with abdominal wall hernia, which draws on related research examining health‐related quality of life and complications for patients following acute symptomatic hernia [42].

In conclusion, this study finds that for patients presenting as emergency hospital admissions with common acute abdominal conditions, emergency and non‐emergency surgery strategies lead to similar average DAOH at 90 days. For patients with severe levels of frailty, emergency surgery strategies may lead to worse outcomes than non‐emergency surgery strategies for each of the five acute conditions. For non‐frail patients, emergency surgery strategies may be more effective for patients with hernia and intestinal obstruction. Further research to inform how best to optimise emergency general surgery provision should recognise that factors such as the patient's frailty level may modify the comparative effectiveness of emergency surgery and non‐emergency surgery strategies.

## Supporting information


**Appendix** **S1.** Section 1. Clinical panel defined inclusion and exclusion criteria.
Section 2. Clinical panel definition of emergency surgery.
Section 3. Tendency to operate as an instrumental variable.
Section 4. Person‐level instrumental variable approach.
Section 5. Proxies for the quality of acute care.
Section 6. Sensitivity analysis.

**Table S1.** Application of ESORT study inclusion and exclusion criteria for emergency admissions to 175 acute NHS hospitals in England April 2010–December 2019.
**Table S2.** Diagnostic subcategories.
**Table S3.** Instrumental variable strength for the hospital‐level tendency to operate within the HES data (2009–19) for emergency admissions that met the ESORT study inclusion criteria for each of the five conditions.
**Table S4.** Clinical management for the emergency surgery and non‐emergency surgery groups in and after (up to 30 days) the time window for emergency surgery.

**Figure S1.** Tendency to operate variation.

**Figure S2.** Balance plots.

**Figure S3.** Kaplan–Meier plots.

**Figure S4.** Mortality forest plots.

**Figure S5.** LOS forest plots.

**Figure S6.** Sensitivity analysis.
