# Supplementary material for: Effectiveness of emergency surgery for five common acute conditions: an instrumental variable analysis of a national routine database
Source: Anaesthesia. 2022 May 19;77(8):865–81. doi: 10.1111/anae.15730 (PMC9540551; doi:10.1111/anae.15730)
Supplement: Supplementary file 1 — Appendix S1. Section 1. Clinical panel defined inclusion and exclusion criteria. Section 2. Clinical panel definition of emergency surgery. Section 3. Tendency to operate as an instrumental variable. Section 4. Person‐level instrumental variable approach. Section 5. Proxies for the quality of acute care. Section 6. Sensitivity analysis. [file ANAE-77-865-s004.docx]

**Appendix S1**

**Section 1: Clinical panel defined inclusion and exclusion criteria**

A clinical panel of 11 surgeons (four women) and one anaesthetist, based in 12 different UK centres across 11 different regions within the UK, that included clinicians with subspecialty interests was convened, and met twice [S1]. A list of ICD10 codes for potential inclusion criteria and potential exclusion diagnoses was presented following the first facilitated meeting and discussed at the second. Each panellist privately rated the potential criteria. A relevant diagnosis for inclusion required at least 75% panel support and diagnoses forming exclusion criteria required at least 25% panel support.

**Section 2: Clinical panel definition of Emergency Surgery**

The panel also defined the procedures that represented ‘emergency surgery’ for patients in each cohort using a Delphi process. A list of Office of Population, Censuses and Surveys classification of surgical operations and procedures (OPCS) codes for potentially relevant procedures for each condition was drawn up and discussed at the first meeting. Panellists indicated privately whether each procedure constituted emergency surgery, and stated the number of days (e.g. three days, five days, seven days etc) within which surgery must be undertaken to count as ‘emergency surgery’. Anonymised panel results were discussed at the second meeting before re-rating. A procedure was defined as constituting ‘emergency surgery’ if according to at least 50% of the panellists the specific procedure met that definition, and it was undertaken within the requisite number of days to meet the criteria for ‘emergency surgery’ according to the median stipulated across the members of the clinical panel (for agreed definition of emergency surgery, see Table S2).

According to the consensus of the clinical panel, the defined time window for emergency surgery was within three days (hernia), seven days (appendicitis, gallstone disease, intestinal obstruction), or any time within the index emergency admission (diverticular disease).

**Section 3: Tendency to Operate (TTO) as an instrumental variable**

The ESORT study uses a preference-based instrumental variable design, with the instrumental variable as the hospital’s Tendency to Operate (TTO) [S2]. The TTO is defined for each qualifying emergency admission, as the proportion of eligible emergency admissions in the 12 months prior to each admission in the specific hospital, who received emergency surgery rather than non-emergency surgery strategies. A precedent study by Keele et al. (2018) took a similar approach, using TTO at the surgeon level, to evaluate emergency surgery versus non-emergency surgery strategies using US claims data [S3]. A recent systematic review identified 185 studies which applied preference based IV methods within health research and reported such methods were most commonly applied to cancer, cardiovascular diseases and mental health [S4]. As noted by the authors, a valid instrumental variable must (A1) strongly predict treatment status (‘relevance’), (A2) affect outcomes only through exposure (‘exclusion’), and (A3) not share any unmeasured causes with the outcome (‘unconfoundedness’) (A4) the IV only affects treatment status in one direction (‘monotonocity’) [S4].

Hospitals that differ in their TTO may be expected to differ in the probability that they will provide emergency surgery to a particular patient. We find considerable variation across hospitals in their TTO, even after adjusting for observable characteristics of the patients. The hospital’s TTO was strongly correlated with emergency surgery receipt for each of the five conditions, even after controlling for a rich set of covariates, with F-statistics that ranged from 450 (diverticular disease) to 24,517 (gallstone disease), see Supplementary Table S6. A commonly applied threshold for ‘weak’ instruments is an F-statistic less than 10 [S5]. Thus, the hospital’s past preference for emergency surgery strongly predicts treatment choice for the current patient.

The validity of the instrumental variable analysis rests on the assumption that, conditional on the included variables, the TTO does not influence the outcomes except through changing the uptake of emergency surgery (assumptions A2 and A3). To increase the plausibility of this assumption, we adjust for fixed effects (indicators) for each financial year, observable patient characteristics, and proxies for hospital quality, a key unobservable confounder. Including hospital fixed effects would result in greatly inflated standard errors since they would remove all between hospital variation. We did not include these fixed effects as they aim to control for time invariant confounders, captured by the proxies for hospital quality (past mortality or readmissions either at baseline or over the preceding year). Moreover, the approach taken, of controlling for baseline and a moving window of mortality and readmissions, reduces the concern that bias may be introduced if sicker patients tend to attend hospitals with a higher (or lower) TTO, since we anticipate that this is captured in the measured covariates including hospital quality. While the assumption that the instrumental variable is not correlated with outcomes or unobserved confounders, conditional on the variables controlled for, is fundamentally untestable, some reassurance is provided if observed covariates do not vary across levels of the instrument. Supplementary Figure S2 illustrates that observed confounders are similar across levels of the TTO, which provides support for the underlying assumptions.

**Section 4:** **Person-level instrumental variable approach**

The instrumental variable approach begins by estimating treatment effects for ‘marginal’ patients, those for whom there was equipoise about the emergency surgery decision according to these measured characteristics (e.g. age, TTO), as well as those unmeasured in the data (e.g. physiology). For these (hypothetical) marginal patients, the instrumental variable approach estimates treatment effects for patients for whom a small change (or nudge) in the TTO (the instrument), can ‘tip the balance’ towards emergency surgery, but does not change the level of any risk factors, including those that are not unmeasured. Comparing outcomes for patients defined according to small differences in the TTO therefore provides an estimate of the causal effect of emergency surgery versus non-emergency surgery for similar patients. By repeating this contrast across different levels of TTO, the study can estimate treatment effects for sets of marginal patients with different combinations of confounders (e.g. frailty levels).

Each individual in the dataset, given their observed and unobserved confounders, would be a marginal patient at *some* level of the TTO. In the absence of further information, we might simply calculate individual-level treatment effects as the average across all marginal patients with similar characteristics to the individual to obtain an effect estimate for each individual. However, the observed decision on whether or not an individual patient has emergency surgery provides some information on the extent to which the levels of unobserved confounders for that individual either encouraged/discouraged emergency surgery. For instance, if an individual who according to the observed baseline measures is at low risk, and presents to a hospital with a low TTO, does actually receive emergency surgery, we can infer their unobserved characteristics were such that they influenced the decision to have emergency surgery. Therefore, for each individual, a treatment effect is obtained by averaging the treatment effects for those marginal patients who share the same observed characteristics, and who has estimated levels of unobserved confounders that are consistent with the observed emergency surgery decision, for that individual, given the TTO in the hospital that they were admitted to, at the time they were first seen by the surgical team (day zero). The estimated individual-level treatment effects can then be averaged over any sample characteristics to report the effectiveness of emergency surgery at the subgroup-level, or for the full sample to obtain an average treatment effect estimate.

This person-level instrumental variable approach was implemented as follows: first, each patient’s propensity for emergency surgery was estimated according to their observed characteristics and the TTO. Second, an outcome model was estimated relating the observed outcome to the individuals’ observed characteristics, and their propensity for emergency surgery, along with interactions between them. After estimating the model, the marginal treatment effects were obtained by considering the impact of a marginal change in the propensity for emergency surgery on outcomes. Third, numerical integration was used to obtain individual level treatment effects. The steps were bootstrapped 300 times (200 times for sensitivity analyses due to computational complexity) to obtain standard errors and confidence intervals. (For further details on the estimation steps, see Basu, 2015) [S6].

**Section 5: Proxies for the quality of acute care**

The validity of an instrumental variable analysis analyses assumes that, conditional on the observed baseline covariates, the instrumental variable does not have a direct effect on the outcome except through influencing the receipt of treatment. The requisite assumptions could be violated if the quality of the acute care, which is an unmeasured variable, was associated with the hospital’s TTO. Information was therefore collated to proxy the quality of acute care for emergency admissions with each acute condition, by extracting from the HES data, rates of all-cause mortality and emergency readmissions up to 90 days for each hospital. The proxy measures for quality were chosen to adjust for both time-constant differences in quality across hospitals, and those that differed over time. This information was reported for each condition for the 2009-2010 financial year, to provide a baseline, time-invariant proxies for care quality in each hospital, and for the 1 year preceding each qualifying emergency hospital admission, to provide time-varying proxies for care quality.

We chose to include these proxies for care quality rather than hospital fixed effects, to provide a more specific proxy for care quality, and to avoid removing all between-hospital variation in rates of emergency surgery which would lead to inflated standard errors in the estimated effectiveness of emergency surgery.

As part of the sensitivity analyses, we considered external measures of hospital quality using data from the National Emergency Laparotomy Audit (NELA) [S7-S9]. Since data was not available for all years of the study and definitions changed over time, we constructed an average (weighted by volume) using data from 2016, 2017 & 2018 for the following seven indicators of quality of peri-operative management for emergency laparotomy patients which we anticipate would capture the influence of any potential time invariant observed confounders associated with hospital quality:

1. Adjusted mortality rate
2. Proportion of patients in whom a risk assessment was documented preoperatively
3. Proportion of patients arriving in theatre within a time appropriate for the urgency of surgery
4. Proportion of patients with a calculated preoperative risk of death >5% for whom a consultant surgeon and anaesthetist were present in theatre
5. Admission to critical care when risk of death ≥5%
6. Unplanned returns to theatre
7. Unplanned returns to critical care

**Section 6: Sensitivity Analyses**

We assessed the extent to which the findings from the main analyses were sensitive to alternative definitions and assumptions. We consider alternative definitions of emergency surgery, by considering a more conservative panel definition of emergency surgery (SA1), and a reduced time window for the procedure to qualify as emergency surgery, by considering only procedures that occurred before the 75th percentile of the time of emergency surgery used in the main analysis (SA2). We explored the sensitivity of the findings to alternative proxies for hospital quality (SA3) by using an external proxy for hospital quality - the NELA quality measures. We also considered the impact of removing hospitals with low volume for the procedures of interest (SA4). We exclude hospitals whose volume of eligible procedures is less that thresholds of one interquartile range below the median, which in this sensitivity analysis led to the exclusion of 22 (Appendicitis), 23 (Gallstone disease), 27 (Diverticular disease) 25 (Hernia) or 16 (Intestinal obstruction) hospitals, and between 2.84% (Intestinal obstruction) and (6.25% Diverticular disease) of observations. We considered alternative definition of the primary outcome, DAOH (SA5), which weights the DAOH prior to death for those who died before day 90 [S10]. Finally, we use regression adjustment to report estimates of relative effectiveness under the assumption of no unobserved confounding (SA6)

**References**

S1. ESORT (2021). Clinical panel. Summary note. <https://www.lshtm.ac.uk/media/39151> [accessed 7^th^ September, 2021]

S2. Brookhart MA, Schneeweiss S. Preference-based instrumental variable methods for the estimation of treatment effects: assessing validity and interpreting results. *The International Journal of Biostatistics* 2007; **3**: article 14.

S3. Fogarty CB, Lee K, Kelz RR, Keele LJ. Biased Encouragements and Heterogeneous Effects in an Instrumental Variable Study of Emergency General Surgical Outcomes. *Journal of the American Statistical Association*, 2021; **116**: 1625-1636.

S4. Widding-Havneraas, T, Chaulagain A, Lyhmann I et al. Preference-based instrumental variables in health research rely on important and underreported assumptions: a systematic review. *Journal of Clinical Epidemiology* 2021; **139**: 269-278.

S5. Staiger D, Stock JH. Instrumental variables regression with weak instruments. *Econometrica* 1997; **65**: 557-586.

S6. Basu A. Estimating Person‐Centered Treatment (Pet) Effects Using Instrumental Variables: An Application to Evaluating Prostate Cancer Treatments. *Journal of Applied Econometrics* 2014; **29**: 671-691

S7. NELA Project Team (2016). Second Patient Report of the National Emergency Laparotomy Audit. RCoA London. Available from <https://www.nela.org.uk/reports> [accessed 7^th^ September, 2021]

S8. NELA Project Team (2017). Third Patient Report of the National Emergency Laparotomy Audit. RCoA London. Available from <https://www.nela.org.uk/reports> [accessed 7^th^ September, 2021]

S9. NELA Project Team (2018). Fourth Patient Report of the National Emergency Laparotomy Audit. RCoA London. Available from <https://www.nela.org.uk/reports> [accessed 7^th^ September, 2021]

S10. Ariti C, Cleland JGF, Pocock SJ, et al. Days alive and out of hospital and the patient journey in patients with heart failure: Insights from the Candesartan in Heart failure: Assessment of Reduction in Mortality and morbidity (CHARM) program. *American Heart Journal* 2011; **162**: 900-906.
