# Supplementary material for: Effectiveness of emergency surgery for five common acute conditions: an instrumental variable analysis of a national routine database
Source: Anaesthesia. 2022 May 19;77(8):865–81. doi: 10.1111/anae.15730 (PMC9540551; doi:10.1111/anae.15730)
Supplement: Supplementary file 2 — Table S1. Application of ESORT study inclusion and exclusion criteria for emergency admissions to 175 acute NHS hospitals in England April 2010–December 2019. Table S2. Diagnostic subcategories. Table S3. Instrumental variable strength for the hospital‐level tendency to operate within the HES data (2009–19) for emergency admissions that met the ESORT study inclusion criteria for each of the five conditions. Table S4. Clinical management for the emergency surgery and non‐emergency surgery groups in and after (up to 30 days) the time window for emergency surgery. [file ANAE-77-865-s005.docx]

**Table S1:** **Application of ESORT study inclusion and exclusion criteria for emergency admissions to 175 acute NHS hospitals in England April 2010-December 2019. Values are number or number (proportion).**

|  | **Appendicitis** | **Gallstone disease** | **Diverticular disease** | **Hernia** | **Intestinal obstruction** |
| --- | --- | --- | --- | --- | --- |
| *Met inclusion criteria* | *307,890* | *365,791* | *200,021* | *146,601* | *236,791* |
| **Exclusions** |  |  |  |  |  |
| No episode with a consultant surgeon | 8582  (2.8%) | 56,913  (15.6%) | 26,606  (13.3%) | 9457  (6.5%) | 34,475  (14.6%) |
| No eligible diagnosis in the first two episodes | 1492  (0.5%) | 5593  (1.5%) | 5501  (2.8%) | 1761  (1.2%) | 7208  (3.0%) |
| Not admitted through A&E or GP | 21,053  (6.8%) | 21,637  (5.9%) | 12,182  (6.1%) | 11,477  (7.8%) | 13,595  (5.7%) |
| Clinical panel exclusion criteria | 1443  (0.5%) | 0  (0.0%) | 0  (0.0%) | 7574  (5.2%) | 10,512  (4.4%) |
| Missing discharge data | 351  (0.1%) | 274  (0.1%) | 146  (0.1%) | 115  (0.1%) | 340  (0.1%) |
| Transfer between hospitals before index episode | 634  (0.2%) | 588  (0.2%) | 406  (0.2%) | 173  (0.1%) | 503  (0.2%) |
| Other admission meeting inclusion criteria in previous 12 months | 3580  (1.2%) | 38,812  (10.6%) | 15,544  (7.8%) | 7908  (5.4%) | 31,686  (13.4%) |
| Emergency surgery prior to index episode | 2518  (0.8%) | 406  (0.1%) | 573  (0.3%) | 842  (0.6%) | 1123  (0.5%) |
| Emergency surgery procedure in prior admission within 90 days | 93  (0.0%) | 591  (0.2%) | 194  (0.1%) | 862  (0.6%) | 4276  (1.8%) |
| *Included in cohort* | *268,144* | *240,977* | *138,869* | *106,432* | *133,073* |

**Table S2: Diagnostic subcategories. Values are number (proportion).**

| **Population** | **ICD-10** | **Description** | **n (%)** |
| --- | --- | --- | --- |
| Appendicitis | K35 | Acute appendicitis | 14,794 (5.5%) |
|  | K35.2 | Acute appendicitis with generalized peritonitis | 10,475 (3.9%) |
|  | K35.3 | Acute appendicitis with localized peritonitis | 59,434 (22.2%) |
|  | K35.8 | Acute appendicitis, other and unspecified | 147,081 (54.9%) |
|  | K37 | Unspecified appendicitis | 36,360 (13.6%) |
|  |  |  |  |
| Diverticular disease | K57.2 | Diverticular disease of large intestine with perforation and abscess | 32,657 (23.5%) |
|  | K57.3 | Diverticular disease of large intestine without perforation or abscess | 106,212 (76.5%) |
|  |  |  |  |
| Gallstone disease | K80.0 | Calculus of gallbladder with acute cholecystitis | 86,878 (36.1%) |
|  | K80.1 | Calculus of gallbladder with other cholecystitis | 67,503 (28.0%) |
|  | K80.2 | Calculus of gallbladder without cholecystitis | 86,596 (35.9%) |
|  |  |  |  |
| Hernia | K40.0 | Bilateral inguinal hernia, with obstruction, without gangrene | 957 (0.1%) |
|  | K40.1 | Bilateral inguinal hernia, with gangrene | 52 (0.0%) |
|  | K40.2 | Bilateral inguinal hernia, without obstruction or gangrene | 1987 (1.9%) |
|  | K40.3 | Unilateral or unspecified inguinal hernia, with obstruction, without gangrene | 17,177 (16.1%) |
|  | K40.4 | Unilateral or unspecified inguinal hernia, with gangrene | 730 (0.7%) |
|  | K40.9 | Unilateral or unspecified inguinal hernia, without obstruction or gangrene | 30,108 (28.3%) |
|  | K41.0 | Bilateral femoral hernia, with obstruction, without gangrene | 263 (0.2%) |
|  | K41.1 | Bilateral femoral hernia, with gangrene | 37 (0.0%) |
|  | K41.2 | Bilateral femoral hernia, without obstruction or gangrene | 55 (0.1%) |
|  | K41.3 | Unilateral or unspecified femoral hernia, with obstruction, without gangrene | 8491 (8.0%) |
|  | K41.4 | Unilateral or unspecified femoral hernia, with gangrene | 1148 (1.1%) |
|  | K41.9 | Unilateral or unspecified femoral hernia, without obstruction or gangrene | 3460 (3.3%) |
|  | K42.0 | Umbilical hernia with obstruction, without gangrene | 18,324 (17.2%) |
|  | K42.1 | Umbilical hernia with gangrene | 1245 (1.2%) |
|  | K42.9 | Umbilical hernia without obstruction or gangrene | 20,235 (19.0%) |
|  | K43.6 | Other and unspecified ventral hernia with obstruction, without gangrene | 2066 (1.9%) |
|  | K43.7 | Other and unspecified ventral hernia with gangrene | 97 (0.1%) |
|  |  |  |  |
| Intestinal obstruction | K56.1 | Intussusception | 1465 (1.1%) |
|  | K56.2 | Volvulus | 16,126 (12.1%) |
|  | K56.3 | Gallstone ileus | 2027 (1.5%) |
|  | K56.5 | Intestinal adhesions [bands] with obstruction | 46,061 (34.6%) |
|  | K56.6 | Other and unspecified intestinal obstruction | 67,394 (50.6%) |

**Table S3:** **Instrumental variable strength for the hospital-level tendency-to-operate (TTO) within the HES data (2009-19) for emergency admissions that met the ESORT study inclusion criteria for each of the five conditions.**

|  | **Montiel-Pflueger robust weak instrument test**  **F-Statistic** |
| --- | --- |
| **Appendicitis** | 141 |
| **Gallstone disease** | 9053 |
| **Diverticular disease** | 206 |
| **Hernia** | 739 |
| **Intestinal obstruction** | 246 |

**Table S4: Clinical management for the emergency surgery and non-emergency surgery groups in and after (up to 30 days) the time window for emergency surgery. Values are number (proportion).**

|  | **Appendicitis** | | | | **Gallstone disease** | | | | **Diverticular disease** | | | | **Hernia** | | | | **Intestinal obstruction** | | | |
| --- | --- | --- | --- | --- | --- | --- | --- | --- | --- | --- | --- | --- | --- | --- | --- | --- | --- | --- | --- | --- |
|  | Emergency surgery  (n=247,506) | | Non-emergency surgery  (n=20,638) | | Emergency surgery  (n=52,004) | | Non-emergency surgery  (n=188,973) | | Emergency surgery  (n=15,772) | | Non-emergency surgery  (n=123,097) | | Emergency surgery  (n=62,559) | | Non-emergency surgery  (n=43,873) | | Emergency surgery  (n=40,550) | | Non-emergency surgery  (n=92,523) | |
| ***In window or after*** | *In* | *after* | *in* | *after* | *in* | *after* | *in* | *after* | *in* | *after* | *in* | *after* | *in* | *after* | *in* | *after* | *in* | *after* | *in* | *after* |
| **Operative**  **procedures**  Emergency surgery procedure  Other procedure^1^  No surgery | 100.0  0.0  0.0 | 0.8  0.1  99.9 | 0.0  15.5  84.5 | 3.4  0.4  96.2 | 100.0  0.0  0.0 | 0.7  2.3  97.0 | 0.0  5.1  94.9 | 9.5  4.1  86.3 | 100.0  0.0  0.0 | 0.5  0.1  99.4 | 0.0  0.1  99.9 | 0.6  0.2  99.2 | 100.0  0.0  0.0 | 0.7  0.2  99.1 | 0.0  12.9  87.1 | 16.5  0.1  83.4 | 100.0  0.0  0.0 | 2.2  0.1  97.7 | 0.0  7.4  92.6 | 5.6  0.1  94.3 |
| **Interventional radiology (IR)**  Abdominal IR  No IR | 0.2  99.8 | 0.4  99.6 | 0.9  99.1 | 0.5  99.5 | 0.4  99.6 | 0.8  99.2 | 0.2  99.8 | 0.5  99.5 | 6.1  93.9 | 0.5  99.5 | 1.5  98.5 | 0.2  99.8 | 0.4  99.6 | 0.7  99.3 | 0.2  99.8 | 0.5  99.5 | 0.9  99.1 | 1.7  98.3 | 0.7  99.3 | 0.6  99.4 |
| **Imaging**  Imaging procedure  No imaging | 27.8  72.2 | 4.0  96.0 | 56.6  43.4 | 8.3  91.7 | 32.2  67.8 | 8.5  91.5 | 37.0  63.0 | 11.0  89.0 | 78.0  22.0 | 4.7  95.3 | 80.6  19.4 | 8.0  92.0 | 16.1  83.9 | 2.3  97.7 | 16.4  83.6 | 1.6  98.4 | 70.1  29.9 | 7.0  93.0 | 54.5  45.5 | 2.4  97.6 |

^1^ If there is no emergency surgery procedure

The table reports the percentage of each sample for the number (n) given under each column heading. There were no missing data’
