# Supplementary figures and images for: Effectiveness of emergency surgery for five common acute conditions: an instrumental variable analysis of a national routine database
Source: Anaesthesia. 2022 May 19;77(8):865–81. doi: 10.1111/anae.15730 (PMC9540551; doi:10.1111/anae.15730)

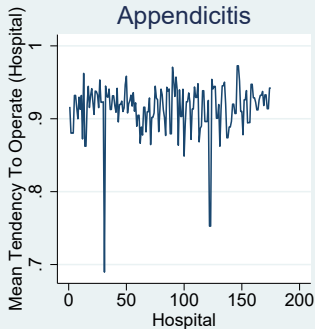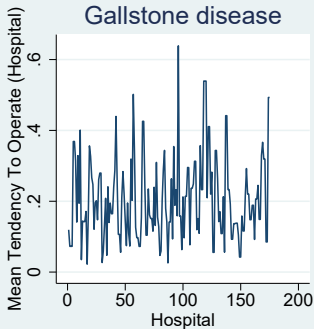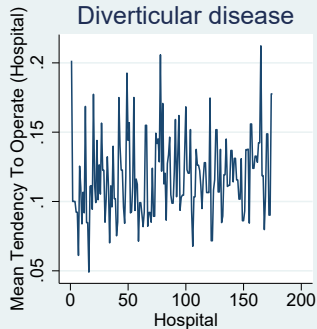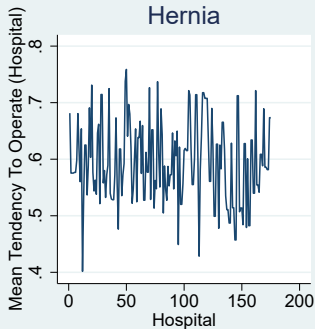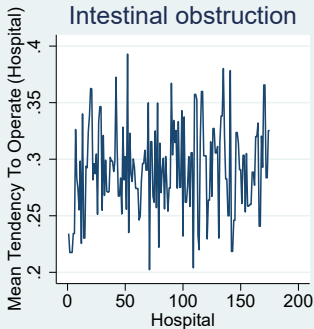

Supplement: Supplementary file 3 — Figure S1. Tendency to operate variation. [file ANAE-77-865-s007.pdf]

Appendicitis

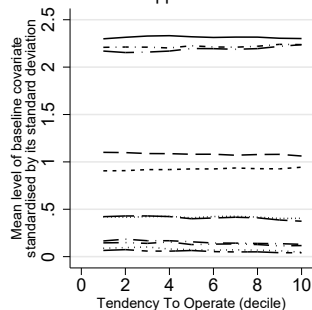

Gallstone disease

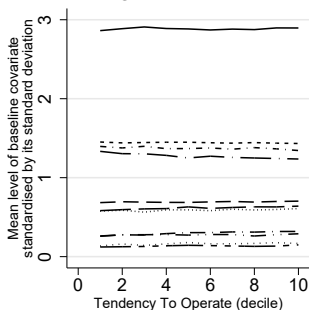

Diverticular disease

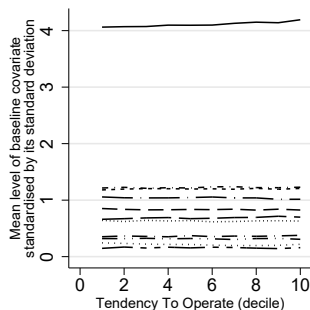

Hernia

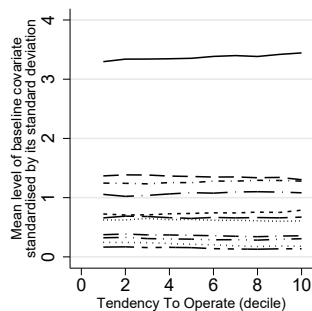

Intestinal obstruction

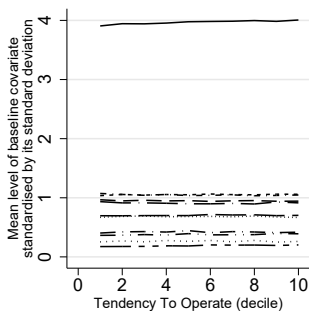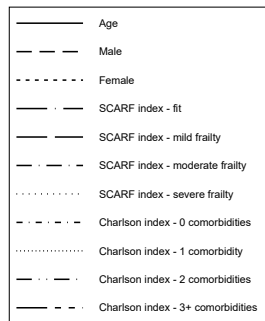

Supplement: Supplementary file 4 — Figure S2. Balance plots. [file ANAE-77-865-s001.pdf]

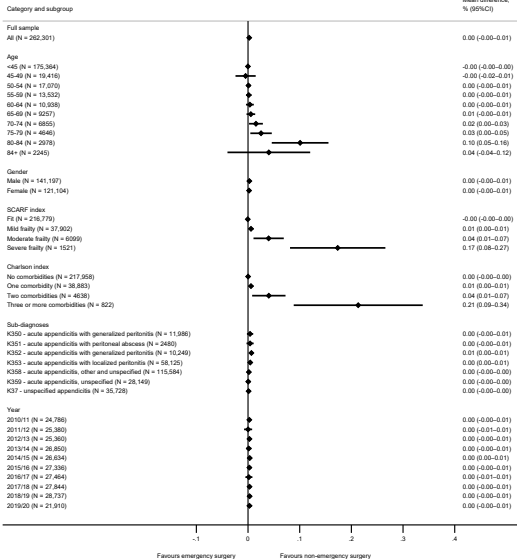

Supplement: Supplementary file 6 — Figure S4. Mortality forest plots. [file ANAE-77-865-s006.zip › anae15730-sup-0006-FigS4a.pdf]

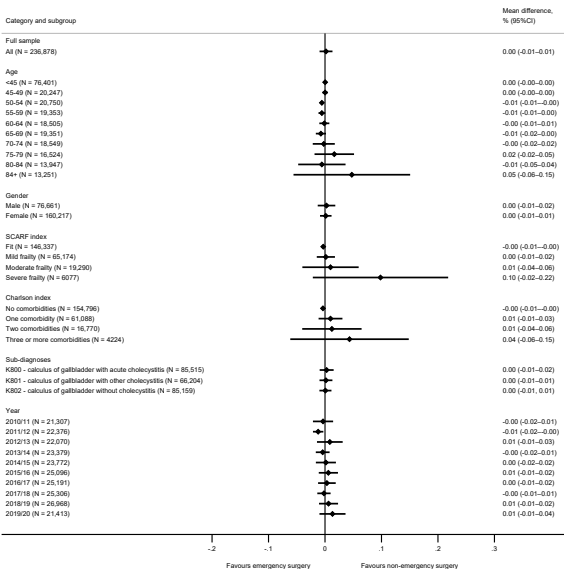

Supplement: Supplementary file 6 — Figure S4. Mortality forest plots. [file ANAE-77-865-s006.zip › anae15730-sup-0007-FigS4b.pdf]

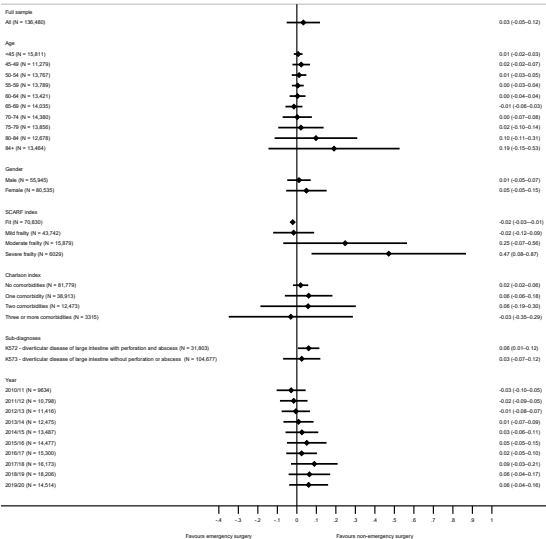

Supplement: Supplementary file 6 — Figure S4. Mortality forest plots. [file ANAE-77-865-s006.zip › anae15730-sup-0008-FigS4c.pdf]

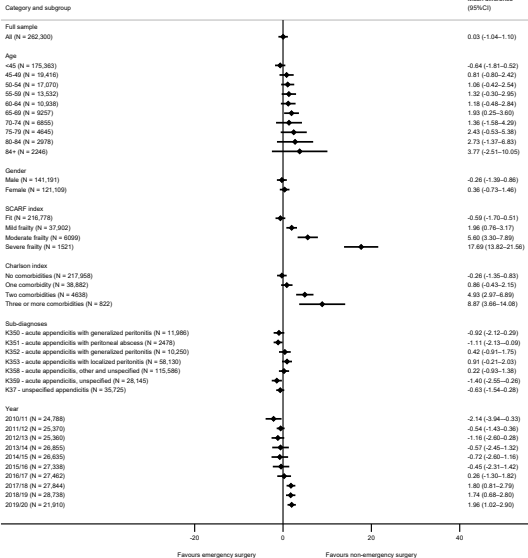

Supplement: Supplementary file 7 — Figure S5. LOS forest plots. [file ANAE-77-865-s003.zip › anae15730-sup-0011-FigS5a.pdf]

## Appendicitis

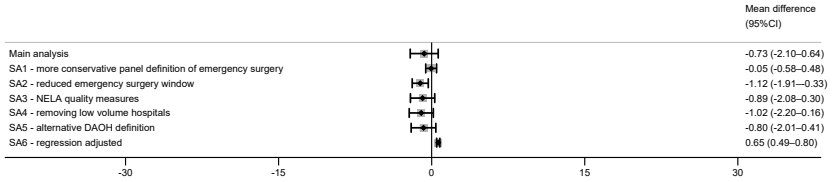

## Gallstone disease

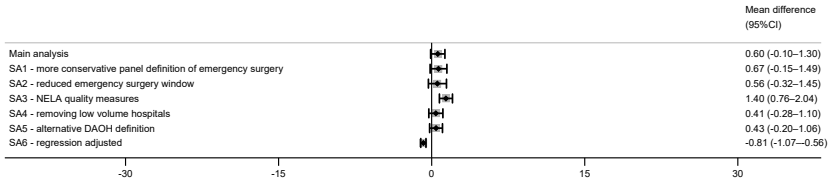

## Diverticular disease

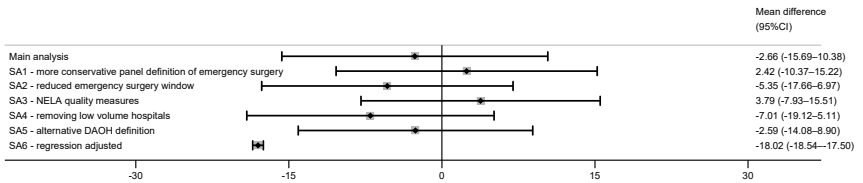

## Hernia

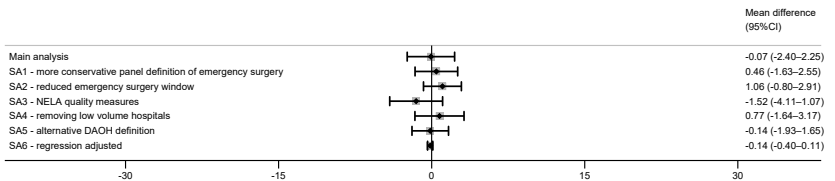

## Intestinal obstruction

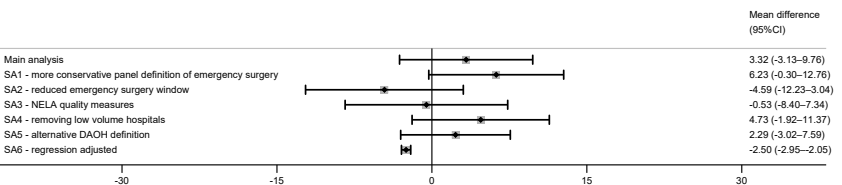

Supplement: Supplementary file 8 — Figure S6. Sensitivity analysis. [file ANAE-77-865-s002.pdf]
