# Supplementary material for: Effectiveness of emergency surgery for five common acute conditions: an instrumental variable analysis of a national routine database
Source: Anaesthesia. 2022 May 19;77(8):865–81. doi: 10.1111/anae.15730 (PMC9540551; doi:10.1111/anae.15730)
Supplement: Supplementary file 5 — Figure S3. Kaplan–Meier plots. [file ANAE-77-865-s008.pdf]

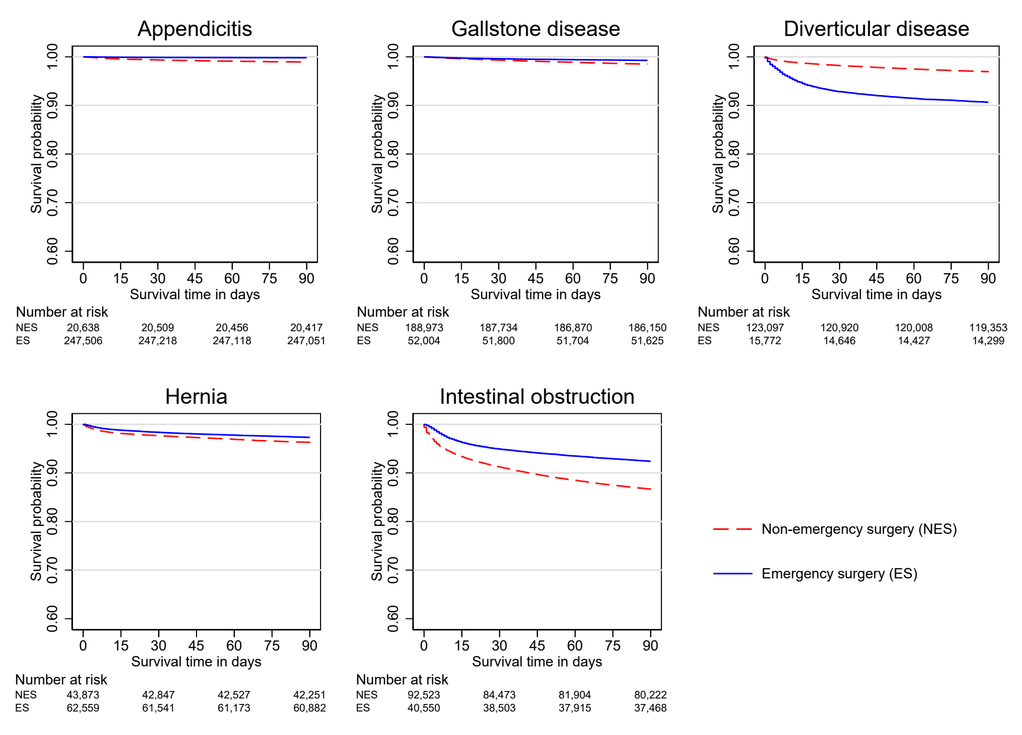
Appendicitis
Survival probability
0.60
0.70
0.80
0.90
1.00
0 15 30 45 60 75 90
Survival time in days
Number at risk
NES 20,638 20,509 20,456 20,417
ES 247,506 247,218 247,118 247,051
Gallstone disease
Survival probability
0.60
0.70
0.80
0.90
1.00
0 15 30 45 60 75 90
Survival time in days
Number at risk
NES 188,973 187,734 186,870 186,150
ES 52,004 51,800 51,704 51,625
Diverticular disease
Survival probability
0.60
0.70
0.80
0.90
1.00
0 15 30 45 60 75 90
Survival time in days
Number at risk
NES 123,097 120,920 120,008 119,353
ES 15,772 14,646 14,427 14,299
Hernia
Survival probability
0.60
0.70
0.80
0.90
1.00
0 15 30 45 60 75 90
Survival time in days
Number at risk
NES 43,873 42,847 42,527 42,251
ES 62,559 61,541 61,173 60,882
Intestinal obstruction
Survival probability
0.60
0.70
0.80
0.90
1.00
0 15 30 45 60 75 90
Survival time in days
Number at risk
NES 92,523 84,473 81,904 80,222
ES 40,550 38,503 37,915 37,468
Non-emergency surgery (NES)
Emergency surgery (ES)
